# Semitendinosus tendons are commonly contaminated with skin flora during graft harvest for anterior cruciate ligament reconstruction

**DOI:** 10.1007/s00167-023-07522-9

**Published:** 2023-08-10

**Authors:** Christoph Offerhaus, Vera Jaecker, Sven Shafizadeh, Leonard Müller, Heiko Hahne, Hilmar Wisplinghoff, Nathalie Jazmati

**Affiliations:** 1 Department of Orthopedic Surgery and Sports Traumatology, Sana Medical Centre, Witten/Herdecke University, Aachener Str. 445-449, 50933 Cologne, Germany; 2 Department of Trauma Surgery, Orthopaedic Surgery and Sports Traumatology, Cologne Merheim Medical Centre, Witten/Herdecke University, Cologne, Germany; 3 Center for Musculoskeletal Surgery, Charité - University Medicine Berlin, Berlin, Germany; 4 Faculty of Medicine, University of Cologne, Cologne, Germany; 5 Wisplinghoff Laboratories, Cologne, Germany; 6 Institute for Virology and Microbiology, Witten/Herdecke University, Witten, Germany; 7 Institute for Medical Microbiology, Immunology and Hygiene, University of Cologne, Cologne, Germany

**Keywords:** Anterior cruciate ligament, Graft harvest, Semitendinosus, Infection prevention, ACL contamination, Vancomycin, Antibiotic resistance

## Abstract

**Purpose:**

To investigate the rate of bacterial contamination of semitendinosus (ST) tendons during graft harvest in anterior cruciate ligament reconstruction (ACLR), in order to precisely specify the underlying pathogens and obtain data on their susceptibility to potential antibiotics.

**Methods:**

In a prospective study, a total of 59 consecutive patients undergoing primary ACLR were recruited from one centre. No patient had history of previous surgery to the knee or showed clinical signs of infection. Four tissue samples of harvested ST tendons for anterior cruciate ligament (ACL) autografts (case group; ST) were examined for evidence of bacterial colonisation and compared to four tissue samples of the native ACL as negative controls (control group; ACL). Three of the respective samples were subjected to cultural microbiological examination and one to 16S rRNA-PCR. Antibiotic susceptibility testing was performed for each pathogen that was identified.

**Results:**

A total of 342 samples were analysed by culture. Significantly more patients showed a positive culture of the ST (33.9%; *n* = 20/59) compared to 3.4% of patients (*n* = 2/59) with positive culturing of the ACL (*p* < 0.0001). Including 16S rRNA-PCR, in a total of 42.4% (25/59) of patients, bacteria were detected in at least one ST sample either by PCR and/or culture. All species found (*n* = 33) belong to the typical skin flora with *Staphylococcus epidermidis* (39.4%; *n* = 13/33) being the most common species, followed by *Staphylococcus capitis* (24.2%; *n* = 8/33). All tested isolates (*n* = 29) were susceptible to vancomycin (29/29, 100%), 69% (*n* = 20/29) to oxacillin and 65.5% (*n* = 19/29) to clindamycin.

**Conclusion:**

ST autografts for ACLR were commonly contaminated with skin commensal bacteria during harvest. One-third of the isolates showed resistance to typical perioperative intravenous antibiotics, whereas all isolates were sensitive to vancomycin. Therefore, routine prophylactic decontamination of all hamstring autografts before implantation should be recommended, preferably with topical vancomycin.

**Level of evidence:**

Level III.

## Introduction

Septic arthritis is a rare but devastating complication following anterior cruciate ligament reconstruction (ACLR) with most studies reporting an incidence around 1.5% [14, 20, 29, 30]. In 2012, Vertullo et al. introduced a technique of presoaking the graft in vancomycin before it is implanted into the knee joint [33]. Clinical studies subsequently have shown a significant reduction of postoperative infection rate after ACLR by this technique [3, 4, 6, 7, 12, 13, 18, 21, 22, 26, 31]. However, at the time this technique was introduced, the exact mechanism of contamination leading to knee joint infection after ACLR was unclear and to some extend still is.

The focus in infection-prevention was specifically directed to the use of hamstring autografts as they were found to be more susceptible to postoperative deep knee infection compared to other auto- and allografts [15–17, 24]. The parameters that explain the vulnerability of hamstring grafts to infection have not been well studied. Bacterial contamination during retrieval and the preparation of the graft are the most accepted hypothesis [1, 8, 11, 25, 34]. Previous studies have focussed on comparison of different grafts or different intraoperative steps of graft harvest and preparation as well as the possibility of reduction/elimination of bacterial contamination of the graft by the use of vancomycin [1, 11, 25, 34]. In addition, microbiological testing is likely to involve iatrogenic contamination during sample collection and/or further processing in the laboratory, which was not controlled in the above studies. Thus, there is still no accurate evidence about the contamination rate of hamstring autografts during harvest for ACLR. To our best knowledge, this is the first study to examine multiple samples at the same time point during the surgery and to compare them to an expectedly uncontaminated control group.

Furthermore, not only evident deep knee infection but also clinically inapparent bacterial colonisation—so-called low-grade infections—appear to be of significant importance for the outcome after ACLR, because they are suspected to be associated with graft failure [22, 23, 26, 28]. Therefore, exact determination of species brought into the knee joint due to bacterial contamination of the graft is essential to derive a therapeutic approach.

The purpose of the present study was to investigate the rate of bacterial contamination of semitendinosus (ST) tendons during graft harvest in ACLR and the precise specification of the underlying pathogens. Results should be compared to simultaneously collected samples of the ruptured native anterior cruciate ligament (ACL) and synovial fluid from knee joint aspiration to estimate percentage of additional iatrogenic contamination during sample collection and/or further processing in the laboratory. In addition, all pathogens found should be tested for their susceptibility to possible antibiotics in order to provide clinical conclusions for possible preventive interventions.

## Materials and methods

Institutional ethics committee approval was obtained prior to the start of this study (ID: 229/2019; University Witten/Herdecke). In a prospective controlled study, tissue samples of harvested ST tendons for ACL grafts (case group; ST) were examined for evidence of bacterial colonisation and compared to tissue samples of the native ACL (control group; ACL) in primary ACLR.

Fifty-nine consecutive patients with primary ACLR with a hamstring tendon autograft were included into study. All patients were recruited from one centre. Written informed consent was obtained from all patients before they were included in the study. Exclusion criteria were preoperative signs or history of knee joint infection, any previous surgery of the injured knee joint, diabetes mellitus, immunosuppressive therapy (e.g. cortisone), malignant disease, chronic inflammatory disease (e.g. Crohn’s disease), age under 18, pregnancy, i.v. drug abuse and antibiotic intake in the past three months. All surgeries were performed at the same medical centre and analysed in the same laboratory between January and December 2021.

### Sampling procedure and surgical protocol

Three days before surgery, a blood sample was taken to determine c-reactive protein (CRP) and white blood cell (WBC) count.

In the operating room, after disinfection of the knee joint and before the start of the actual procedure, a knee joint puncture was performed to obtain synovial fluid. The fluid was transferred to a sterile vessel for microbiological examination. If no fluid could be obtained, a “punctio sicca” was documented.

Skin antisepsis was performed with the repeated application of an alcoholic solution. After draping and before starting the surgical procedure, all surgical staff changed gloves. Diagnostic arthroscopy was performed at the beginning of the procedure. After confirmation of the diagnosis of an ACL rupture, ACL remnants were harvested right at the beginning of the arthroscopy and divided into four tissue samples of equal size. A minimum sample size of 5 × 5 mm was requested in all cases to allow further analysis. Three of these tissue samples were subjected to cultural microbiological examination and one to 16S rRNA-PCR.

After diagnostic arthroscopy, ST graft harvest was performed in a standard technique using an open hamstring graft harvester. During retrieval of the tendon, contact to the patients’ skin and the surgeon’s gloves was avoided as much as possible using a sterile gauze. The tendon was cut and transferred to the side-table where four samples were immediately collected with previously unused instruments and without prior debridement or cleaning of the tendon. In a recent study, Wu et al. demonstrated that the contamination rate after graft harvest did not differ depending on whether the samples were taken at the ends or in the middle of the graft [34]. Therefore, in the present study, samples were randomly taken from the graft as best suited for the surgical procedure that followed. A minimum sample size of 5 × 5 mm was requested in all cases to allow further analysis. Three of these tissue samples were subjected to cultural microbiological examination and one to 16S rRNA-PCR.

After sample collection, the harvested tendon was “dipped” into a 5 mg/ml vancomycin solution prepared as described by Grayson et al. [10]. After the graft was prepared, it was wrapped in a gauze saturated with this solution. Prior to transplantation, the graft was washed with 20 ml of sterile saline.

The tissue and synovial samples were transported from the operating room to the laboratory by a certified transport service of the laboratory immediately after surgery, or at the latest within four hours after collection. Samples were processed in the laboratory immediately after receipt.

### Laboratory analysis

All samples for microbiological and molecular analysis were processed at Wisplinghoff Laboratories (Cologne, Germany) according to the German microbiological diagnostic standard for the diagnostic of joint infections [27]. All laboratory methods used were accredited by the national accreditation body of the Federal Republic of Germany (DAKKS) and validated accordingly.

### Microbiological methods

Biopsies were homogenised using sterilised 7 ml Precellys lysing Kits CK28 (Bertin Technologies, France) prefilled with 3 ml NaCl. The homogenised sample was used to perform Gram stain and was streaked out onto Columbia agar supplemented with 5% sheep blood, chocolate agar and Schaedler agar (Becton Dickinson, US). In addition, for enrichment 1 ml of the homogenised sample was inoculated, respectively, into an aerobe and anaerobe blood culture bottle (BCB) (BACT/Alert FA plus and SN, bioMérieux, France), using a sterile syringe. Aerobic agar plates were incubated at 37° ± 1 °C under aerobic conditions. Schaedler blood agar was incubated in an aerobic jar system (Anoxomat II, Mart Microbiology, Netherlands) also at 37 ± 1 °C. BCBs were incubated in the BACT/Alert 3D instrument (bioMerieux, France) and incubation time was set to 14 days.

Isolated bacteria were identified according to the standard laboratory procedures (i.e. MALDI-TOF (Bruker, US) and phenotypic tests). Antibiotic susceptibility testing was performed by Microscan Walkaway plus System (Beckman Coulter, US) and interpreted according to EUCAST v.12.

### Molecular methods

DNA extraction was performed using Ultra-Deep Microbiome Prep Kit (Molzym, Germany) and 16s RNA realtime-PCR was performed by commercially available CE-marked Ultra-Clean Bacterial DNA Assay (Molzym, Germany).

Sanger sequencing of 16s RNA-PCR positive samples was performed after purification of PCR products via GeneticAnalyzer 3130 XL (Applied Biosystems, US). The consensus sequences were compared with those in the BLAST database. All tests were performed according to the manufacturer’s instructions.

### Statistical analysis

Descriptive statistics including median and standard deviation were calculated for all recorded variables. Single missing values were not included in the analysis. Differences between the two groups were tested by Chi-square test and Fishers exact test. A *p-*value of less than 0.05 (two-tailed) was considered statistically significant. All statistical analyses were performed using SPSS (v.28, IBM corp.). To estimate the required sample size, we modelled the following situation: in a paired analysis with a two-tailed one degree-of-freedom Chi-square test, alpha error of 0.05 and beta error of 0.2, assuming 17% of contamination in the ST group (based on previous studies [1, 11, 25]) versus 0% in the ACL group, a total of 43 study participants was required. Post hoc power analysis showed that the detection methods applied lead to higher effect sizes and thus, together with the 59-person sample, to an actual power of more than 98%.

## Results

In total, 59 patients with primary ACLR using a quadrupled ST graft were included into the study. Mean age was 32 ± 2 years and 67.8% of patients were male. Further characteristics of the study population are shown in Table 1.

**Table 1:** Study population and clinical findings

	All patients (*n* = 59)			
	*n*	%	Mean	SD
Patients’ characteristics				
Age	59		32	2
Sex				
M	40	67.8		
W	19	32.2		
Height (m)	59		1.78	0.08
Weight (kg)	59		83.7	2.3
BMI	59		26.4	0.7
Preoperative blood analysis				
WBC count/µl	59		6.7	1.6
CRP mg/dl				
< 0.4	52	88.1		
0.5	3	5.1		
0.6	3	5.1		
1	0	0.0		
1.2	1	1.7		

*WBC* White blood cell, *BMI* body mass index, *SD* standard deviation

### Microbiological analysis

A total of 165 samples from ST grafts and 177 samples from the torn ACL from 59 patients were included in the microbiological analysis. In addition, we included 27 samples of synovial fluid aspirations for cultural analysis. Upon culturing, significantly more bacteria were detected in the ST samples (*n* = 26/165; 15.8%) compared to samples from the ACL (*n* = 4/177; 2.3%) (*p* < 0.0001) as well as compared to samples from the synovial fluid (0/27; 0%) (*p* = 0.0267). This was also true on a patient level: 33.9% of patients (*n* = 20/59) showed a positive culture of the ST compared to 3.4% of patients (*n* = 2/59) with positive culturing of the ACL (*p* < 0.0001).

These results were confirmed by 16 s-RNA-PCR which was available for 49 patients: semitendinosus samples were positive in 12.2% (6/49), while in the ACL group, only 1 out of 49 samples (2.0%) was positive by PCR (n.s.).

Even with Gram staining, which is known to be the less sensitive analytic method for detecting bacteria, we found differences between the groups: 2 of 165 samples (1.2%) had Gram-positive cocci in the ST group compared to 0% in the ACL group (0/177) (n.s.) and compared to 0% (0/27) in the synovial fluid group (n.s.). All details of the microbiological analysis are shown in Table 2.

**Table 2:** Comparison of microbiological and laboratory results of semitendinosus, native ACL and synovial samples

	Semitendinosus (*n* = 165 biopsies)		Native ACL (*n* = 177 biopsies)		Synovial fluid (*n* = 27)		p (semitendinosus vs. ACL)	p (semitendinosus vs. synovial fluid)	p (ACL vs. synovial fluid)
	*n*	%	*n*	%	*n*	%			
Cultural analysis									
Sterile	139	84.2	173	97.7	27	100.0%	** < 0.0001**	** < 0.0001**	n.s
Growth	26	15.8	4	2.3	0	0.0%			
PCR (16s RNA)									
Negative	43	87.8	48	98.0	N/A	N/A	n.s	N/A	N/A
Positive	6	12.2	1	2.0	N/A	N/A			
Gram stain									
Negative	163	98.8	177	100.0	27	100.0%	n.s	n.s	n.s
Positive	2	1.2	0	0.0	0	0.0%			

Significant results are shown in bold

*N/A* Not applicable, *n.s*. not significant

Overall, bacteria were detected in at least one ST sample by either PCR and/or culture in 42.4% (25/59) of patients compared to only 5.1% (3/59) of ACL samples (*p* < 0.0001). All patients with positive results in culture and/or PCR are shown in detail in Table 3.

**Table 3:** Microbiological and laboratory results of all patients with detection of bacteria

Patient	Microbiological analysis semitendinosus tendon (directly after explantation)				Cultural analysis native ACL (microbiology)				
	Culture semitendinosus 1	Culture semitendinosus 2	Culture semitendinosus 3	PCR (16s RNA) semitendinosus	Culture biopsy 1	Culture biopsy 2	Culture biopsy 2	Synovial fluid culture	PCR (16s RNA)
Only semitendinosus samples positive									
001	N/A	*Staphylococcus caprae*	N/A	0	Sterile	Sterile	Sterile	N/A	0
002	*Staphylococcus hominis*	N/A	N/A	0	Sterile	Sterile	Sterile	N/A	0
004	*Staphylococcus epidermidis*	N/A	N/A	N/A	Sterile	Sterile	Sterile	Sterile	N/A
007	sterile	N/A	N/A	*Staphylococcus epidermidis*	Sterile	Sterile	Sterile	Sterile	0
008	*Staphylococcus capitis*	Sterile	Sterile	*Staphylococcus epidermidis*	Sterile	Sterile	Sterile	Sterile	0
010	Sterile	Sterile	Sterile	*Lactobacillus acidophilus*	Sterile	Sterile	Sterile	Sterile	0
014	Sterile	Sterile	Sterile	*Staphylococcus *spp*. (schleiferi, aureus, chromogenes)*	Sterile	Sterile	Sterile	Sterile	0
018	*Staphylococcus epidermidis*	Sterile	Sterile	0	Sterile	Sterile	Sterile	N/A	0
020	*Staphylococcus capitis*	*Staphylococcus capitis*	Sterile	0	Sterile	Sterile	Sterile	N/A	0
025	Sterile	*Staphylococcus haemolyticus*	Sterile	0	Sterile	Sterile	Sterile	N/A	0
041	Sterile	*Staphylococcus epidermidis*	Sterile	0	Sterile	Sterile	Sterile	N/A	0
047	Sterile	*Staphylococcus epidermidis*	*Staphylococcus epidermidis*	0	Sterile	Sterile	Sterile	Sterile	0
051	*Staphylococcus epidermidis*	Sterile	Sterile	0	Sterile	Sterile	Sterile	Sterile	0
056	*Staphylococcus capitis, Staphylococcus aureus*	*Staphylococcus epidermidis*	Sterile	N/A	Sterile	Sterile	Sterile	N/A	N/A
057	Sterile	Sterile	Sterile	*Staphylococcus epidermidis*	Sterile	Sterile	Sterile	N/A	0
060	*Staphylococcus epidermidis*	Sterile	Sterile	0	Sterile	Sterile	Sterile	Sterile	0
062	*Staphylococcus hominis*	Sterile	Sterile	N/A	Sterile	Sterile	Sterile	Sterile	N/A
074	Sterile	*Micrococcus luteus*	Sterile	0	Sterile	Sterile	Sterile	Sterile	0
077	Sterile	*Staphylococcus saprophyticus*	Sterile	0	Sterile	Sterile	Sterile	N/A	0
081	Sterile	*Staphylococcus saccharolyticus*	Sterile	0	Sterile	Sterile	Sterile	Sterile	0
082	Sterile	Sterile	*Staphylococcus hominis*	0	Sterile	Sterile	Sterile	N/A	0
084	Sterile	Paracoccus spp.	Sterile	0	Sterile	Sterile	Sterile	Sterile	0
109	*Staphylococcus hominis*	Sterile	Sterile	N/A	Sterile	Sterile	Sterile	Sterile	N/A
ACL and semitendinosus samples positive									
076	*Staphylococcus epidermidis*	*Staphylococcus capitis, Staphylococcus epidermidis*	*Staphylococcus capitis, Staphylococcus epidermidis*	Negative	*Staphylococcus capitis*	*Staphylococcus capitis*	*Staphylococcus epidermidis*	N/A	Negative
089	Sterile	Sterile	*Staphylococcus epidermidis*	*Staphylococcus hominis*	Sterile	Sterile	Sterile	N/A	*Corynebacterium tuberculostearicum*
Only ACL samples positive									
023	Sterile	Sterile	Sterile	Negative	*Staphylococcus saccharolyticus*	Sterile	Sterile	N/A	Negative

*N/A* Not applicable as sample/result is missing

In total, 33 isolates were detected by culture in our study. *Staphylococcus epidermidis* (*n* = 13/33; 39.4%) was the most common species, followed by *Staphylococcus capitis* (*n* = 8/33; 24.2%). All species belong to the typical skin flora. A detailed distribution of the species can be found in Table 4.

**Table 4:** Distribution of species found by culture

Species	Number of isolates	%
*Staphylococcus epidermidis*	13	39
*Staphylococcus capitis*	8	24
*Staphylococcus hominis*	4	12
*Staphylococcus saccharolyticus*	2	6
*Micrococcus luteus*	1	3
*Paracoccus *spp*.*	1	3
*Staphylococcus caprae*	1	3
*Staphylococcus haemolyticus*	1	3
*Staphylococcus saprophyticus*	1	3
*Staphylococcus aureus*	1	3
Total	**33**	**100**

Significant results are shown in bold

Likewise, *Staphylococcus epidermidis* was the most common bacterium found by PCR (*n* = 3/7; 42.9%). Details are found in Table 5.

**Table 5:** Distribution of species found by 16s RNA-PCR

Species	Number of isolates	%
*Staphylococcus epidermidis*	3	43
*Corynebacterium tuberculostearicum*	1	14
*Lactobacillus acidophilus*	1	14
*Staphylococcus hominis*	1	14
*Staphylococcus *spp*. (schleiferi, aureus, chromogenes)*	1	14
Total	**7**	**100**

Significant results are shown in bold

Of the 33 isolates, 29 could be tested for susceptibility to vancomycin, oxacillin and clindamycin. For *Staphylococcus saccharolyticus *(*n* = 2), *Micrococcus luteus *(*n* = 1) and *Paracoccus *spp*.* (*n* = 1), no breakpoints for the interpretation according to EUCAST exist for these antibiotics. Thus, these isolates were excluded from susceptibility test analysis.

All tested isolates were susceptible to vancomycin (29/29; 100%). To clindamycin, 65.5% (*n* = 19/29) were susceptible (s), 10.3% (*n* = 3/29) susceptible at increased exposure (i) and 24.1% (*n* = 7/29) resistant (r). Of the isolates tested, 31% (*n* = 9/29) were resistant to oxacillin (and consequently to first- and second-generation cephalosporins).

## Discussion

The main finding of the present study was that ST tendon autografts for ACLR are frequently contaminated with skin commensal bacteria during graft harvest. All species detected were susceptible to the use of vancomycin.

Hantes et al. were the first to investigate contamination rates of two different autografts (bone–patellar tendon–bone and hamstrings) in 60 cases of ACLR [11]. In their study, three tissue samples were obtained for culture from each graft at different time-intervals during graft preparation. Overall, bacterial presence was comparable between the two grafts: 13% for hamstring autografts and 10% for bone–patellar tendon–bone grafts, with the contamination rate increasing during graft preparation.

In a similar setting, Gavriilidis et al. investigated a consecutive series of 89 patients with hamstring autograft ACLR [9]. In their study which focussed on the correlation of contamination of the graft with clinical infection and its association with C-reactive protein (CRP) and erythrocyte sedimentation rate (ESR), they collected two tissue samples from each graft for culture. Although no clinical infection was present, 11 samples from 9 patients (10%) had positive culture results.

Pérez-Prieto et al. reported that contamination of bone–patellar tendon–bone and hamstrings autografts occurred during graft harvest and preparation and could be eradicated by the use of topical vancomycin [25]. In a case series including 50 patients from 3 different hospitals, they collected 1 tissue sample after graft harvest and 2 samples after graft manipulation and preparation. One of the latter two samples was soaked in vancomycin. In their series, bacterial contamination of the graft was observed in 4% of the cases after graft harvest and in 14% of the cases after graft preparation. This contamination could fully be eradicated after soaking the last sample in vancomycin.

Alomar et al. compared contamination rate of hamstring grafts after preparation with samples that were intentionally dropped onto the operating room floor [1]. In 50 patients, rates of positive cultures were almost as high in hamstring autografts (22%) as in samples dropped on the floor (32%).

Compared to these previous studies [1, 9, 11, 25], the present investigation showed the highest contamination rate of harvested ST tendons with the cultural detection of bacteria in 33.9% of patients. In our opinion, this is mainly due to the fact that the present study was the first to examine multiple tissue samples at the same intraoperative time point. This procedure of multiple sampling is microbiological standard to increase the sensitivity of the culture [27]. Considering only the rate of positive tissue samples in relation to the total number of samples collected, our investigation showed a positive rate of 15.8%. Although these results are not absolutely comparable, they appear to be consistent with the above studies and clearly demonstrate the effect of increased sensitivity due to multiple sampling. Therefore, we believe that contamination rate was underestimated in previous studies.

Including 16s-RNA-PCR, even 42.4% of patients were tested positive for bacteria in the present study. Nevertheless, care should be taken when interpreting PCR-results. Studies reported variable sensitivity of this method and concluded that this PCR technique should just be used as a complement to culture to help clarify confusing culture results in selected samples [2, 5, 19].

Interestingly, in the patients with positive culture samples and positive 16s rRNA-PCR, none of the species found in culture were confirmed by PCR. Instead, different species were detected, that did not match the cultural result. The reason for this might be that cultivation and PCR were performed from different samples. However, this also underlines that the bacteria detected are random contaminants from the skin flora of the patient and possibly also of the surgical staff. The fact that the contamination rate in the comparison group (native ACL) was significantly lower (3.4% of patients with positive culturing, *p* < 0.0001) allows to exclude an accidental contamination during sample collection and further processing in the laboratory and shows that these contaminations occurred during graft harvest and thus are of significant importance.

Despite the high contamination rate, no knee joint infections were detected in 1 year of follow-up. This observation is consistent with previous studies in which deep knee infections were also not observed despite the high contamination rates of the harvested grafts [9, 11]. The risk of developing a deep knee infection after microbial contamination of the surgical site is multifactorial, depending besides the virulence of the pathogen and patient immunity on the level of contamination, measured by CFU. Alomar et al. demonstrated that level of contamination after graft harvest and preparation was below the threshold of 10^5^ CFU/g of tissue, which is associated with an elevated risk of surgical wound infection [1]. However, hamstring grafts typically require the use of sutures, buttons or possibly screws for fixation, which increases the risk of infection even at low levels of contamination. Furthermore, not only evident deep knee infection but also clinically inapparent bacterial colonisation—so-called low-grade infections—appear to be of significant importance for the outcome after ACLR, because they are suspected to be associated with graft failure [22, 26]. Therefore, routine prophylactic decontamination of all hamstring autografts prior to implantation is recommended [32].

All bacteria detected in the present study belong to the typical skin flora. *Staphylococcus epidermidis* (39.4%) was the most common species, followed by *Staphylococcus capitis* (24.2%). This is coherent with the findings of previous studies and the fact that deep knee infections after ACLR are mainly associated with CNS [1, 9, 11, 15, 21, 22]. Interestingly, this high prevalence of CNS is not seen in other acute orthopaedic-related infections [35]. For this reason, possible contamination with skin flora during graft harvest and manipulation is considered a major risk factor for deep knee infections after ACLR.

Susceptibility testing showed resistance in one-third of the isolates to first and second-generation cephalosporins, which are the main antibiotics used for perioperative intravenous (IV) prophylaxis. Similarly, 24% of isolates were resistant to clindamycin, which is commonly used as a second-line perioperative IV antibiotic. All species were susceptible to vancomycin which is coherent with the findings of Pérez-Prieto et al. who reported that bacterial contamination from grafts could be completely eliminated by topical application of vancomycin [25]. Thus, clinical studies showed that the postoperative infection rate after ACL reconstruction could be significantly reduced to almost 0% by soaking the hamstring tendon graft in vancomycin before it was implanted into the knee joint [3, 4, 6, 7, 12, 13, 18, 21, 22, 26, 31].

The present study had several limitations: no further samples were taken before implantation of the graft. Considering that only a small portion of the ST tendon was examined immediately after harvest and that previous studies showed increasing contamination from graft preparation [9, 11, 25], an even higher presence of bacteria on the implanted graft must be assumed. After sample collection and graft preparation, the graft was soaked in vancomycin as this is standard surgical protocol at the research centre’s institution to prevent septic arthritis after ACRL. Therefore, no statement can be made whether the high number of contaminations would also have led to infections. Furthermore, assessment of postoperative infection was based on a phone interview at minimum 1 year after surgery. Low-grade infections and subclinical infection signs such as prolonged pain, persistent effusion and stiffness may have been overlooked by this method.

## Conclusion

The results of the present study demonstrate that ST autografts for ACLR are commonly contaminated with skin commensal bacteria, with one-third showing resistance to typical perioperative IV antibiotics. All isolates were sensitive to vancomycin, which from this point of view is another reason for its standard topical application on the graft after harvest and before implantation.
